# 2D medical image synthesis using transformer-based denoising diffusion probabilistic model

**DOI:** 10.1088/1361-6560/acca5c

**Published:** 2023-05-05

**Authors:** Shaoyan Pan, Tonghe Wang, Richard L J Qiu, Marian Axente, Chih-Wei Chang, Junbo Peng, Ashish B Patel, Joseph Shelton, Sagar A Patel, Justin Roper, Xiaofeng Yang

**Affiliations:** 1 Department of Radiation Oncology and Winship Cancer Institute, Emory University, Atlanta, GA 30322, United States of America; 2 Department of Biomedical Informatics, Emory University, Atlanta, GA 30322, United States of America; 3 Department of Medical Physics, Memorial Sloan Kettering Cancer Center, New York, NY 10065, United States of America; 4 Nuclear and Radiological Engineering and Medical physics Programs, George W. Woodruff School of Mechanical Engineering, Georgia Institute of Technology, Atlanta, GA 30332, United States of America

**Keywords:** transformer-based diffusion model, Swin-transformer-based network, medical image synthesis, COVID-19, artificial intelligence

## Abstract

*Objective*. Artificial intelligence (AI) methods have gained popularity in medical imaging research. The size and scope of the training image datasets needed for successful AI model deployment does not always have the desired scale. In this paper, we introduce a medical image synthesis framework aimed at addressing the challenge of limited training datasets for AI models. *Approach*. The proposed 2D image synthesis framework is based on a diffusion model using a Swin-transformer-based network. This model consists of a forward Gaussian noise process and a reverse process using the transformer-based diffusion model for denoising. Training data includes four image datasets: chest x-rays, heart MRI, pelvic CT, and abdomen CT. We evaluated the authenticity, quality, and diversity of the synthetic images using visual Turing assessments conducted by three medical physicists, and four quantitative evaluations: the Inception score (IS), Fréchet Inception Distance score (FID), feature similarity and diversity score (DS, indicating diversity similarity) between the synthetic and true images. To leverage the framework value for training AI models, we conducted COVID-19 classification tasks using real images, synthetic images, and mixtures of both images. *Main results*. Visual Turing assessments showed an average accuracy of 0.64 (accuracy converging to $50 \% $ indicates a better realistic visual appearance of the synthetic images), sensitivity of 0.79, and specificity of 0.50. Average quantitative accuracy obtained from all datasets were IS = 2.28, FID = 37.27, FDS = 0.20, and DS = 0.86. For the COVID-19 classification task, the baseline network obtained an accuracy of 0.88 using a pure real dataset, 0.89 using a pure synthetic dataset, and 0.93 using a dataset mixed of real and synthetic data. *Significance*. A image synthesis framework was demonstrated for medical image synthesis, which can generate high-quality medical images of different imaging modalities with the purpose of supplementing existing training sets for AI model deployment. This method has potential applications in many data-driven medical imaging research.

## 1. Introduction

Deep learning (DL) has been actively investigated and increasingly applied in medical physics. While DL methods have the advantage of being less task-dependent and generalizable, they rely on large, representative datasets for training and are therefore prone to challenges in their clinical implementation. Accumulating appropriately sized patient data training sets for a specific disease can take in excess of multiple years if the disease incident rate is small. Even when large datasets are available, raw data pre-processing, a crucial step for model stability and precision in various medical tasks such as organ segmentation, radiomic-based predictions, reinforcement learning, etc (Lei *et al* 2020a, Lei *et al* 2020b, Liu *et al* 2020, Song *et al* 2021, Guo *et al* 2021, Dai *et al* 2021b, Pan *et al* 2022b, Hu *et al* 2022, Chang *et al* 2022, Pan *et al* 2022a), requires a lot of human efforts for collecting data. Furthermore, storing and sharing large amounts of patient data can raise data security and privacy concerns. Current mitigation strategies include conventional image augmentation such as scaling, rotation, affine and deformed transformations on existing training datasets, thus providing very limited intrinsic diversity of the dataset. Therefore, better data augmentation methods are needed. To address these challenges, a medical image synthesis methods were proposed (Ho *et al* 2020). Synthetic data can supplement conventional data augmentation when large-scale data is needed for deep learning training, and it can be used for a variety of other purposes, such as imaging system design and development, virtual clinical trials, and statistical model development. The synthetic data should be similar in morphology and texture to real data and add greater diversity in visual appearances and data characteristics to enrich existing datasets.

DL based medical image synthesis methods have been recently proposed, specifically including generative adversarial network (GAN) (Goodfellow *et al* 2020). While GANs have achieved state-of-the-art results in various tasks (Yi *et al* 2019, Kazeminia *et al* 2020, Pan *et al* 2021), including image creation (Salehinejad *et al* 2019, Pan *et al* 2021, Zunair and Hamza 2021), image super-resolution (Yamashita and Markov 2020, Dai *et al* 2021a), and image-to-image translation (Lei *et al* 2019, Armanious *et al* 2020, Wang *et al* 2021, Amirrajab *et al* 2022), they inevitably suffer from unstable training and mode collapse (Arjovsky *et al* 2017), which can affect the quality and diversity of the generated synthetic images. To address these limitations, diffusion models were proposed as alternative to GANs, capable to generate better quality and more diverse synthetic images (Ho *et al* 2020, Dhariwal and Nichol 2021, Nichol and Dhariwal 2021). Diffusion models utilize a neural network (typically a U-shape convolutional neural network (U-net) (Ronneberger *et al* 2015)) to learn denoising in order output visually-appealing medical images. Diffusion models can avoid the adversarial training typically needed in GANs, thus improving training stability and generating more authentic images (Dhariwal and Nichol 2021). Within the scope of medical images, Wolleb *et al* (2022) proposed an implicit diffusion model to translate normal or ‘healthy’ chest x-rays into ‘unhealthy’ chest x-rays as a data augmentation step, in order to expand their training set for their proposed anomaly detection algorithm. Herein, we aim to introduce a generalized diffusion model framework for 2D medical data synthesis that works across imaging modalities. To our knowledge, this is a first in training set augmentation. Furthermore, the proposed Transformer-based Denoising Diffusion Probabilistic Model (MT-DDPM) is the first to incorporate Swin-vision transformer (Liu *et al* 2021). This generalized diffusion model implementation could also be further developed for other image synthesis tasks, such as image-to-image translation.

Novel to diffusion model implementation, we proposed a U-Swin-transformer network to perform the 2D denoising (diffusion) process. In medical image denoising, U-net has demonstrated consistent accuracy (Nasrin *et al* 2019, Jia *et al* 2021). U-net architecture typically consists of a convolutional encoder and decoder. The encoder is a down-sampling path to encode the input images into features of different resolutions. Moreover, the decoder is a de-compression path that up-samples the extracted features to assemble the final denoised image. Although U-net can achieve good performance, they suffer from limited ability to model global-level dependency from input scans in most specific dataset (Karras *et al* 2017), limiting the denoising accuracy. The vision transformer (VIT) (Liu *et al* 2021), designed based on a self-attention mechanism (Vaswani *et al* 2017), has a solid capability for long-range model dependency, and has been introduced to further boost denoising performance. Therefore, the proposed U-Swin-transformer network can capture both global and local information effectively to perform a high-quality diffusion process. We propose a Swin-transformer-based medical diffusion model (MT-DDPM) for 2D medical image synthesis. The model consists of a forward process for generating noisy images and a reverse process for denoising them. By applying the diffusion process multiple times, the network can denoise an altered image to a synthetic image, new to the dataset. The proposed network has an encoder–decoder architecture that follows the framework of V-net. Novel to the network design, the Swin-transformer layer is deployed with the convolutional layer to capture both the local-level and global-level information, thus further improving the synthesis quality. We evaluated the model’s ability to generate realistic images from four imaging modalities using four independent MT-DDPMs, respectively: (1) Chest x-rays from 300 patients from the public NIH Chest x-rays dataset (Wang *et al* 2017). (2) Heart MRIs’ 2D slices from 101 patients comprising the ACDC dataset (Bernard *et al* 2018). (3) Male pelvic CT 2D slices in an institutional dataset collected from 123 patients. (4) Abdomen CT 2D slices from public abdominal dataset (Landman *et al* 2015) released by MICCAI collected from 30 patients. The synthetic images were analyzed using visual Turing assessment and quantitative evaluation, including Fréchet Inception Distance score (FID), Inception score (IS), and feature distribution similarity. The framework was also applied it to generate artificial lung CT images and used them in a classification task for COVID-19 diagnosis to validate its effectiveness in actual medical-AI practice. Finally, we conducted an ablation study to examine the impact of different hyper-settings on the model performance.

## 2. Method

**Figure 1.**
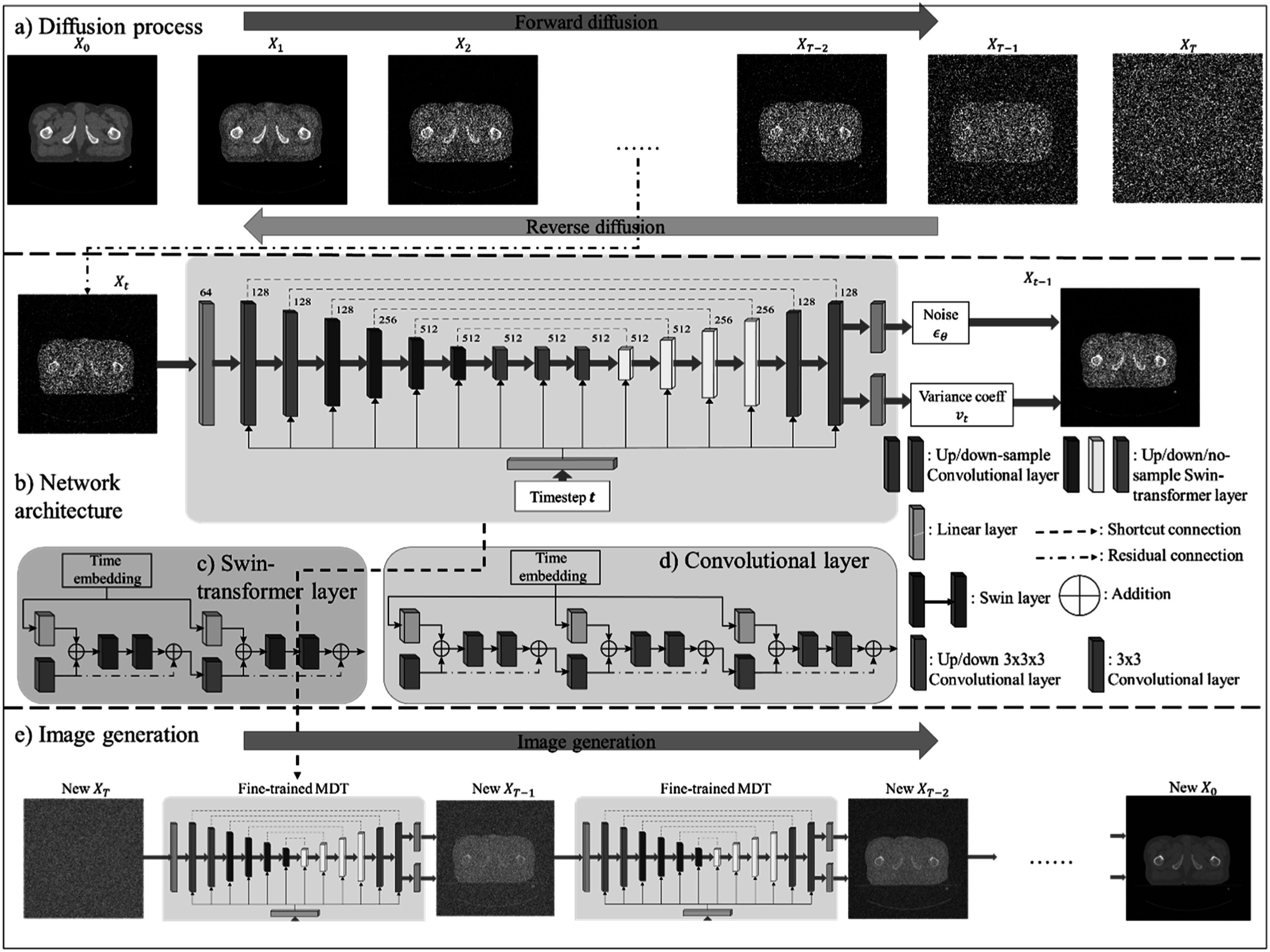
(a) Diffusion process of the MT-DDPM framework: The forward diffusion transforms medical images into pure Gaussian noise by adding a small amount of noise iteratively. The reverse process requires a network to repeatedly denoise the Gaussian noise to the noise-free image. (b) Network architectures of the MT-DDPM network: a symmetrical encoder–decoder architecture to learn the reverse process. We can calculate the denoised image by predicting the noise and variance coefficient by equation (23). (c) Swin-transformer block: the purple Swin layer is built by a window self-attention and a shifted window self-attention module. (d) Convolutional block: Three convolutional residual structures learn local features. (e) Image generation: the fine-training MT-DDPM network can transform a new pure Gaussian noise image into a synthetic image new to the dataset.

The TMD runs a diffusion process to transfer a two-dimensional Gaussian noisy sample ${X}_{T}\sim {\mathscr{N}}\left(0,1\right)$ into a 2D synthetic medical image $X.$ The diffusion process is motivated by an important assumption: we can overlay small amount of noise ${\epsilon }$ to any medical image $X$ in $t\,$timesteps to convert the $X$ to a pure Gaussian noise sample $T,$ with sufficiently large $t.$ Accordingly, the noise sample $T$ can be converted back to the noise-free image $X$ by removing the overlaid noise. In the diffusion process (figure 1(a)), the proposed MT-DDPM network is designed to accurately estimate a noise ${{\epsilon }}_{t}$ and a variance interpolation coefficient at any arbitrary timestep $t,$ which can gradually remove the noise ${\epsilon }$ in multiple timesteps until reconstructing the noise-free image $X.$ Once the MT-DDPM can accurately denoise all the images from a known dataset, the network can finally reconstruct the new high-quality medical image ${X}_{new}$ by denoising a new Gaussian noisy sample ${T}_{new}.$

As illustrated in figure 1(a)), MT-DDPM-net employs a two-dimensional vision transformer encoder–decoder architecture inherited from a token-based vision transformer (Pan *et al* 2022a, 2022b) and Swin-Unet (Cao *et al* 2021). First, the encoder (left) is a down-sampling path consisting of two residual convolutional blocks and four Swin-transformer-based blocks to learn compressed features from noisy input data ${X}_{t}\,\in {{\mathbb{R}}}^{H\times W},$ where $H$ and $W$ are length and width of ${X}_{t}.$ Then the decoder (right) is an up-sampling path, which has a symmetrical structure to the encoder, decompresses the features to estimate the noise ${{\epsilon }}_{t}\in {{\mathbb{R}}}^{H\times W}$ and a variance interpolation coefficient ${{\mathrm{v}}}_{{\mathrm{t}}}\in {{\mathbb{R}}}^{H\times W}$ at timestep $t.$ Here we introduce the MT-DDPM -net’s architecture and the diffusion process’s mathematical formulation.

### 2.1. Network architecture

The full architecture is shown in figure (b). In the encoder, the input medical images are first passed into a convolutional layer with a kernel size of $3\times 3$ and stride 1 to learn early features. The features are then down-sampled by a factor of 2 in each block. For the encoder’s architecture, we propose first to have two down-sampled convolutional blocks of learning early local characteristics from inputs with relatively high resolutions. Then, we apply four sequential down-sampled Swin-transformer blocks to learn global information from features of low resolutions. Then, three middle Swin-transformer (without down-sampling or up-sampling) blocks are connected to further calculate global characteristics. Finally, the decoder consists of four up-sampled Swin-transformer blocks with two final up-sampled convolutional blocks to recover the features back to their original resolution. The final features are passed into two convolutional layers: one estimates the noise, and the other estimates the variance interpolation coefficient. In addition, the timestep is encoded by sinusoidal embedding (SE) (Ho *et al* 2020) (maximum period of SE is selected as ${10}^{6},$ feature dimension is selected as 128). The timestep embeddings are then input into all the blocks for further calculations.

As shown in figures (c) and (d), we take advantage of the ‘Residual connection’ (He *et al* 2016) to further improve the network stability. In addition, as shown in figure (b), across the blocks, we utilize the ‘shortcut connection’ (Ronneberger *et al* 2015) to connect each encoder block to a decoder block in the same resolution level to better convey high-resolution information from the encoder to the decoder and enhance the estimation accuracy.

Regarding network training, we implemented an AdamW optimizer (Loshchilov and Hutter 2017) with a learning rate of 0.000 02 and weight decay of 0.0001. The network was trained in 250 epochs and a batch size of 8.

#### 2.1.1. Convolutional block

Each up/down-sampling convolutional block combines three sequential residual convolutional sections. Each section is built by a timestep expanding (linear) layer, an early convolutional layer, and two subsequent residual layers. Each convolutional layer has $3\times 3$ spatial filters with stride 1 in all axes. In the last section, a trilinear up/down-sampling interpolation is deployed before the early convolutional layer. As shown in figure 1(b)), the early convolutional layer accepts the input features to learn local semantic information.

On the other hand, the timestep expanding layer re-embeds the timestep embeddings to align with the convolutional layer’s dimension. The outputs of the early convolutional layer and the re-embedded timesteps are summed up, then passed into the subsequent convolutional layers. Such that each convolutional section can learn semantic information conditioning on timestep $t.$ A residual connection is applied between the early convolutional layer and the final output. A group normalization (Wu and He 2018) (number of groups is 32) and a SiLu (Elfwing *et al* 2018) activation function is applied to every convolution layer.

#### 2.1.2. Swin-transformer block

The Swin-transformer block has a similar architecture with the convolutional block: two sequential Swin-transformer sections. Each section also consists of a timestep expanding layer, which re-embeds the timestep embedding, and an early $3\times 3$ convolutional layer, which learns local semantic information from input features. Finally, their outputs are summed up and fed to the following Swin-transformer block, which consists of window-attention (WA) and shifted window-attention (S-WA) modules (Liu *et al* 2021), to capture global information.

As shown in figure 1(c)), WA and S-WA modules consist of a window-partition layer to divide the input features $F$ into non-overlapping windows. Then a multi-head self-attention (MHSA) layer, where each head indicates a set of independent weight matrices $Q,\,K,\,V,$ is applied to calculate the spatial information within each window. Practically, we split the input feature $F\in {{\mathbb{R}}}^{H\times W\times C}$ into ${F}_{split}\in {{\mathbb{R}}}^{\displaystyle \frac{H}{l}\times \displaystyle \frac{W}{l}\times ({l}^{2}\times C)},$ to obtain $\displaystyle \frac{H}{l}\times \displaystyle \frac{W}{l}$ 2D windows with size of ${l}^{2}\times C.$ For each window $W,$ the MHSA layer performs:

\begin{eqnarray*}hea{d}_{m}=softmax\left(\displaystyle \frac{W{Q}_{m}{\left(W{K}_{m}\right)}^{T}}{\sqrt{d}}\right)\left(W{V}_{m}\right)\end{eqnarray*}

\begin{eqnarray*}T=Concat\left(hea{d}_{1},\ldots ,hea{d}_{M}\right)O\end{eqnarray*}

where ${Q}_{m},\,{K}_{m},\,{V}_{m}$ are the weights matrices of the ${m}^{{\prime} }th$ head, $O$ is another weight matrices, $T\in {{\mathbb{R}}}^{{l}^{2}\times C}$ is the attention output from the window. By gathering the attention from all the windows, we can finally acquire an attention map ${T}_{all}\in {{\mathbb{R}}}^{\displaystyle \frac{H}{l}\times \displaystyle \frac{W}{l}\times ({l}^{2}\times C)}$ and reshaped back to the original size of the non-split feature $F.$ A fully-connected layer is then applied to further refine the attention map.

The attention map is then input to the S-WA layer. The W-SA layer is identical to the SA layer, which shifts the window by $\left[\tfrac{l}{2},\tfrac{l}{2}\right]$ in the x and *y*-axis. Accordingly, the Swin-transformer section can also learn attention across different windows, better capturing global-level information and improving network performance. In summary, the Swin-transformer layer performs the following:

\begin{eqnarray*}{F}_{w}=linear\left(WA\left(F\right)\right)\end{eqnarray*}

\begin{eqnarray*}Y=linear\left(S-WA\left({F}_{w}\right)\right)\end{eqnarray*}

where $linear$ is fully-connected layer.

### 2.2. Diffusion process

Following (Ho *et al* 2020), we formulate the diffusion-based medical image synthesis process in three stages: (1) a forward diffusion process that gradually overlays small amount Gaussian noise *T* successive timesteps to a given source medical image ${X}_{0},$ to generate a sequence of noisy image $\left\{{X}_{0},\,{X}_{1},\ldots ,{X}_{T}\right\}.$ (2) a reverse diffusion process, learned by the proposed MT-DDPM network, removes the small amount overlayed noises at each timestep, so recursively denoises ${X}_{T}$ to the original image ${X}_{0}.$ (3) Given the MT-DDPM can denoise ${X}_{T}\,$back to the corresponding ${X}_{0}$ regardless of any source image ${X}_{0}$ from an existing medical dataset, a new Gaussian noise sample ${X}_{Tnew}$ is fed to the MT-DDPM network and reverted to a synthetic image ${X}_{new}.$ In summary, the diffusion process demonstrates a deep-learning framework:

1. The forward diffusion process will generate a large number of noisy images as training data.
2. Then, the proposed MT-DDPM, is trained as a denoiser by utilizing the training data, learns a reverse diffusion process to denoise the noisy images.
3. The optimal MT-DDPM can recursively denoise new Gaussian noise back to the noise-free images, so generate synthetic image new to the existing dataset.

#### 2.2.1. Forward diffusion

In forward diffusion, we define the noisy image generation process $q$ as a Markov process which that the noisy image at timestep $t$ only depends on the noisy image at timestep $t-1:$

\begin{eqnarray*}q\left({X}_{t}| {X}_{t-1}\right)={\mathscr{N}}\left({X}_{t};\sqrt{1-{\beta }_{t}}{X}_{t-1},{\beta }_{t}I\right),\end{eqnarray*}

where ${\mathscr{N}}$ defines a normal distribution and ${\beta }_{t}$ is variances at timestep $t.$ Motivated by the reparameterization trick (Ho *et al* 2020), we are able to efficiently represent noisy images at any arbitrary timestep $t$ by

\begin{eqnarray*}{\alpha }_{t}\,:=1-{\beta }_{t}\end{eqnarray*}

\begin{eqnarray*}q\left({X}_{T}| {X}_{0}\right)={\mathscr{N}}\left({X}_{t};\sqrt{\displaystyle \prod _{i=1}^{t}{\alpha }_{i}}{X}_{0},\left(1-\displaystyle \prod _{i=1}^{t}{\alpha }_{i}\right)I\right).\end{eqnarray*}

Practically, the noisy image at timestep *t* is generated as

\begin{eqnarray*}{X}_{t}=\,\left.\sqrt{\displaystyle \prod _{i=1}^{t}{\alpha }_{i}}{X}_{0}+\sqrt{1-\displaystyle \prod _{i=1}^{t}{\alpha }_{i}}{{\epsilon }}_{t}\right),\end{eqnarray*}

where ${{\epsilon }}_{t}\sim {\mathscr{N}}\left(0,\,I\right)$ is a noise sampled from a normal distribution. The maximum timestep *T* is chosen as 4000; and we linearly schedule the variance as ${\beta }_{t}=\,5{e}^{-6}t.$

#### 2.2.2. Reverse diffusion

The reverse diffusion aims to calculate the conditional probability $q\left({X}_{t-1}| {X}_{t}\right).$ Then, we can denoise a random Gaussian noise ${X}_{T}$ to ${X}_{T-1}$ recursively in reverse direction until we are able to generate a corresponding medical image ${X}_{0}.$ The conditional probability can be calculated in a closed form only when conditioned on ${X}_{0}:$

\begin{eqnarray*}\mu \left({X}_{t},{X}_{0},t\right):=\displaystyle \frac{\sqrt{1-{\prod }_{i=1}^{t-1}{\alpha }_{i}}}{1-{\prod }_{i=1}^{t}{\alpha }_{i}}{X}_{0}+\displaystyle \frac{\sqrt{{a}_{t}}(1-{\prod }_{i=1}^{t-1}{\alpha }_{i})}{1-{\prod }_{i=1}^{t}{\alpha }_{i}}{X}_{t},\end{eqnarray*}

\begin{eqnarray*}{\mathrm{\Sigma }}\left({X}_{t},t\right):=\,\displaystyle \frac{1-{\prod }_{i=1}^{t-1}{\alpha }_{i}}{1-{\prod }_{i=1}^{t}{\alpha }_{i}}{\beta }_{t},\end{eqnarray*}

\begin{eqnarray*}q\left({X}_{t-1}| {X}_{t},{X}_{0}\right)={\mathscr{N}}\left({X}_{t-1};\mu \left({X}_{t},{X}_{0},t\right),\,{\mathrm{\Sigma }}\left({X}_{t},t\right)\right).\end{eqnarray*}

However, in our medical image synthesis, the ${X}_{0}$ should be a new unknown image to the existing dataset. Following (Song *et al* 2020) , we propose a neural network $\theta $ to learn a probability ${p}_{\theta }\left({X}_{t-1}| {X}_{t}\right),$ without necessary of conditioning on ${X}_{0},$ to efficiently approximate the $q\left({X}_{t-1}| {X}_{t},{X}_{0}\right),$ which is therefore represented as

\begin{eqnarray*}{p}_{\theta }\left({X}_{t-1}| {X}_{t}\right)={\mathscr{N}}\left({X}_{t-1};{\mu }_{\theta }\left({X}_{t},t\right),\,{{\mathrm{\Sigma }}}_{\theta }\left({X}_{t},t\right)\right),\end{eqnarray*}

where ${\mu }_{\theta }$ is a mean matrix and ${{\mathrm{\Sigma }}}_{\theta }$ is a variance matrix of the approximated Gaussian distribution learned by the network $\theta .$ Once the MT-DDPM learns ${p}_{\theta }\left({X}_{t-1}| {X}_{t}\right)$ for all the ${X}_{0}$ existing in the training dataset, by using the reparameterization trick again, we can recursively generate denoised image ${X}_{t-1}^{new}$ until obtaining the new ${X}_{0}^{new}\,$without the needs of conditioning on ${X}_{0}^{new}:$

\begin{eqnarray*}{X}_{t-1}^{new}={\mu }_{\theta }\left({X}_{t}^{new},t\right)+{\sigma }_{\theta }\left({X}_{t}^{new},t\right)\,\ast \,{{\epsilon }}_{t}\end{eqnarray*}

where ${{\epsilon }}_{t}$ is a random Gaussian noise sampled from ${\mathscr{N}}\left(0,I\right).$ In conclusion, the proposed MT-DDPM network is trained to estimate the mean and standard deviation with given input ${X}_{t}$ and timestep $t.$

##### 2.2.2.1. Estimating the mean

Directly estimate the mean $\mu $ by the MT-DDPM could be the most obvious option. However, learning $\mu $ can be a difficult task for the MT-DDPM, since the value of the mean is not constrained, which causes the training to be unstable and therefore detrimental the synthetic image’s quality. Alternatively, to improve the network’s stability and synthetic image’s quality, we optimize the MT-DDPM as a noise predictor, as shown in figure 1(a), instead of a direct mean predictor.

Practically, as demonstrated in (Ho *et al* 2020), the first output of MT-DDPM , ${{\epsilon }}_{\theta },$ can be optimized by a mean square error (MSE) loss between the ground truth noise ${{\epsilon }}_{t}$ and predicted noise ${{\epsilon }}_{\theta }\left({X}_{t},t\right):$

\begin{eqnarray*}\mathop{{\mathrm{argmin}}\,}\limits_{{{\epsilon }}_{\theta }}{{\mathrm{L}}}_{{\mathrm{mean}}}=MSE\left({{\epsilon }}_{t},{{\epsilon }}_{\theta }\left({X}_{t},t\right)\right)=\,E\left({E}_{t}{\left|\left|{{\epsilon }}_{t}-{{\epsilon }}_{\theta }\left({X}_{t},t\right)\right|\right|}_{2}^{2}\right),\end{eqnarray*}

where $E$ indicates the mean of the square error over all pixels, ${E}_{t}$ indicates the mean over all timesteps. Once an accurate ${{\epsilon }}_{\theta }$ is obtained, we can calculate the mean ${\mu }_{\theta }:$

\begin{eqnarray*}{\mu }_{\theta }\left({X}_{t},t\right)=\displaystyle \frac{1}{\sqrt{{\alpha }_{t}}}\left({X}_{t}-\displaystyle \frac{{\beta }_{t}}{\sqrt{1-{\alpha }_{t}}}{{\epsilon }}_{\theta }\left({X}_{t},t\right)\right)\end{eqnarray*}

Since then, we can obtain the ${\mu }_{\theta }\left({X}_{t},t\right)$ which can be used for the new image synthesis.

##### 2.2.2.2. Estimating the variance

Regarding to the variance, following Nicol *et al*’s demonstration (Dhariwal and Nichol 2021), the ${{\mathrm{\Sigma }}}_{\theta }$ can be calculated by the pre-scheduled variance ${\beta }_{t}$ and an interpolation coefficient $v$ predicted by the MT-DDPM :

\begin{eqnarray*}{{\mathrm{\Sigma }}}_{\theta }\left({X}_{t},t,\,{v}_{t}\right)=\exp \left({v}_{t}\,\ast \,\mathrm{log}\,{\beta }_{t}+\left(1-{v}_{t}\right)\,\ast \,\mathrm{log}\left(\displaystyle \frac{1-{\prod }_{i=1}^{t-1}{\alpha }_{i}}{1-{\prod }_{i=1}^{t}{\alpha }_{i}}{\beta }_{t}\right)\right)\end{eqnarray*}

The predicted variance ${{\mathrm{\Sigma }}}_{\theta }$ should minimize a variational lower bound (VLB) between the ${p}_{\theta }\left({X}_{t-1}| {X}_{t}\right)$ and $q\left({X}_{t-1}| {X}_{t},{X}_{0}\right).$

Practically, we summarize the VLB loss (Nichol and Dhariwal 2021) at timestep $t$ as:

\begin{eqnarray*}{{\mathrm{argmin}}}_{v}\,{{\mathrm{L}}}_{{\mathrm{var}}}=E\left({E}_{t}\left({{\mathrm{L}}}_{{\mathrm{VLB}}}\left({\mathrm{t}}\right)\right)\right)\end{eqnarray*}

And the details of the VLB loss is summarized as:

\begin{eqnarray*}\begin{array}{l}{L}_{t}:=\,\displaystyle \frac{1}{2}\left(\mathrm{log}\,{{\mathrm{\Sigma }}}_{\theta }\left({X}_{t},t,\,{v}_{t}\right)-\,\mathrm{log}\,{\mathrm{\Sigma }}\left({X}_{t},t\right)\right.\\ \,+\left.\displaystyle \frac{\mathrm{log}\,{\mathrm{\Sigma }}\left({X}_{t},t\right)}{\mathrm{log}\,{{\mathrm{\Sigma }}}_{\theta }\left({X}_{t},t,\,{v}_{t}\right)}+\displaystyle \frac{{\left({\mu }_{\theta }\left({X}_{t},t\right)-\mu \left({X}_{t},t\right)\right)}^{2}}{{{\mathrm{\Sigma }}}_{\theta }\left({X}_{t},t,\,{v}_{t}\right)}\right),\end{array}\end{eqnarray*}

\begin{eqnarray*}{L}_{high}:=\left({{\mathrm{\Sigma }}}_{\theta }\right.\left({X}_{t},t,\,{v}_{t}\right)\,\ast \,GELU\left({X}_{t}-{\mu }_{\theta }+\delta \right),\end{eqnarray*}

\begin{eqnarray*}{L}_{low}:=\left({{\mathrm{\Sigma }}}_{\theta }\right.\left({X}_{t},t,\,{v}_{t}\right)\,\ast \,GELU\left({X}_{t}-{\mu }_{\theta }-\delta \right),\end{eqnarray*}

\begin{eqnarray*}{{\mathrm{L}}}_{{\mathrm{VLB}}}\left({\mathrm{t}}\right)=\left\{\begin{array}{c}{L}_{t},\,t> 0\\ \mathrm{log}({L}_{high}-{L}_{low}),\,t=0\,and\,1-\delta < {X}_{t}< \delta \\ \mathrm{log}(1-{L}_{low}),\,t=0\,and\,{X}_{t}> \delta \\ \mathrm{log}({L}_{high}),\,t=0\,and\,{X}_{t}< 1-\delta \end{array}\right.\end{eqnarray*}

where $\delta $ is the pixel intensity threshold set to $\displaystyle \frac{1}{256}.$ The $GELU$ (Gaussian Error Linear Units function) is a fast approximation of Cumulative Distribution Function for Gaussian Distribution (Hendrycks and Gimpel 2016). Notice that there is only one learnable parameter ${v}_{t}$ in the equation and the other parameters are known. Once an optimal ${v}_{t}$ is obtained, we can obtain the ${{\mathrm{\Sigma }}}_{\theta }$ for the image synthesis. The overall optimization function is presented as

\begin{eqnarray*}L={L}_{mean}+\gamma {L}_{var},\end{eqnarray*}

where $\gamma $ is a weighting parameter which is empirically selected as 0.01.

##### 2.2.2.3. Synthetic image generation

Once a trained MT-DDPM can optimally predict the noise ${{\epsilon }}_{\theta }$ and the variance interpolation coefficient ${v}_{t},$ we are able to transform a new Gaussian noise image to synthetic medical data using the MT-DDPM network. As the efficient generation algorithm shown in (Nichol and Dhariwal 2021), we evenly spaced numbers between 1 and $4000$ (the training timestep T) by 500 timesteps and denote the new number set as $S.$ By denoting the new Gaussian noisy sample as ${X}_{s}\,,$ the MT-DDPM can generate a less noisy image ${X}_{s-1}$ by equations (4), (7) and (8) and all we need is to provide ${{\epsilon }}_{s}\sim {\mathscr{N}}\left(0,I\right)$

\begin{eqnarray*}\begin{array}{l}{X}_{s-1}=\,\displaystyle \frac{1}{\sqrt{1-{\beta }_{s}}}\left({X}_{s}-\displaystyle \frac{{\beta }_{s}}{\sqrt{1-\overline{{\alpha }_{s}}}}{{\epsilon }}_{\theta }\left({X}_{s},s\right)\right)\\ +\exp \left({v}_{s}\,\ast \,\mathrm{log}\,{\beta }_{s}+\left(1-{v}_{s}\right)\,\ast \,\mathrm{log}\left(\displaystyle \frac{1-{\prod }_{i=1}^{s-1}{\alpha }_{i}}{1-{\prod }_{i=1}^{s}{\alpha }_{i}}{\beta }_{s}\right)\right)\,\ast \,{{\epsilon }}_{s}\end{array}\end{eqnarray*}

By recursively generate the ${X}_{s-1}$ until we obtain the noise-free image ${X}_{0},$ which is a synthetic medical image new to the existing dataset.

## 3. Database

The MT-DDPM proposed in this study was evaluated using four datasets. We trained different models for each dataset. In each experiment, all images of the dataset were used for training. Then the new synthetic images were used for evaluation.

### 3.1. Public dataset: chest x-rays dataset

The chest x-rays dataset for our synthesis was built from the NIH Chest x-rays dataset (Wang *et al* 2017), which consists of 112120 chest x-rays 2D images saved in PNG format. The first 5500 x-rays images were selected to train the MT-DDPM network. Each training image was then resampled to resolution of $256\times 256.$ For training, the whole dataset was normalized to intensity range from $\left[0,255\right]$ to $[-1,1].$ For inference, the intensities of the synthetic images were clipped to $[-1,1].$

### 3.2. Public dataset: heart MRI dataset

We collected 1902 2D heart MRIs from 3D MR scans of the Automated Cardiac Diagnosis Challenge (ACDC) dataset (Bernard *et al* 2018). The MR scans were collected as a series of axial slices cover the heart base to the left ventricle with an axial spacing of $5$ to 8 millimeters (mm). The 2D axial slices were then separated to form the training dataset. The axial slices of all scans were resampled to $2\times 2$ mm, and boundary-padded to have a consistent resolution of $256\times 256.$ In training, the intensities of each scan were normalized to $[-1,1].$

### 3.3. Institutional dataset: pelvic CT dataset

The dataset contains 4157 2D CT slices collected from 94 patients with prostate cancer treated by external beam radiation therapy in our institution. Institutional review board approval was obtained, and informed consent was not required for this Health Insurance Portability and Accountability Act compliant retrospective analysis. All patient scans were acquired by CT simulation using a Siemens SOMATOM Definition AS CT scanner with a resolution of $512\times 512\times 128$ and voxel size of $0.977\times 0.977\times 2$ mm. The scans were then resampled to voxel size of $2\times 2\times 2$ mm. To reduce irrelevant body volumes, we only collect the central axial slices, covering the bladder, prostate, rectum, left femoral head, and right femoral head. The slice collection was guided by the patient organ segmentation map identified by two expert physicians. The slices were boundary-padded to has a consistent resolution of $256\times 256.$ For training, the pixel intensities of all scans were jointly normalized from attenuation coefficient values $[-1024,3012]$ to $[0,1].$ For inference, the intensities of synthetic images were clipped to $[0,1]$ and converted back to attenuation coefficient values.

### 3.4. Public dataset: abdomen CT dataset

The dataset contains 2178 abdomen CT slices collected from the Beyond the Cranial Vault (BTCV) dataset (Landman *et al* 2015), published in the proceedings of the 2015 Medical Image Computing and Computer-Assisted Intervention conference in 2015. The dataset contains 30 patient scans with a resolution of $512\times 512\times [80\unicode{x00303}225]$ and voxel size of $0.76\times 0.76\times 3$ mm. The patient scans were firstly resampled to a voxel size of $1.52\times 1.52\times 3$ mm and hence each axial slice has resolution of $256\times 256.$ With the guidance of the provided organ annotations, we only collected the slices with at least one organ appearing. The same normalization and attenuation coefficient value recovering strategies adopted in the institutional pelvic CT dataset were implemented in training and inference.

## 4. Implementation and performance evaluation

### 4.1. Implementation details

The MT-DDPM framework and the competing networks were implemented on a workstation running Windows 11 with a 48 Gigabytes NVIDIA RTX 6000 GPU. The experiments were implemented using the Pytorch framework in Python 3.8.11.

### 4.2. Visual turing assessment

Visual Turing assessment, conducted by three American Board of Radiology certified medical physicists, were performed between the real and synthetic images to evaluate the quality of synthetic images. All physicists independently provided their results. They were asked to identify which image was real and which was synthetic from a dataset mixed with real and synthetic images, without knowing the ratio of real/synthetics images given to them. We generated 25 image samples from each implemented dataset, including Chest x-rays, Heart MRI, pelvic CT image, and abdomen CT image; 25 real images were randomly selected from each dataset to mix with the synthetic images. The average accuracy, sensitivity, and specificity of the group were presented. Accuracy values of $50 \% $ represent random guess from the observers, which indicates a more realistic visual appearance of the synthetic images among the originals.

### 4.3. Quantitative comparison

On the other hand, we performed two comprehensive evaluations, an intensity distribution evaluation, and a diversity evaluation, to quantify the quality of the 400 synthetic images generated by the MT-DDPM. In terms of comprehensive evaluations, the Inception score (IS) (Salimans *et al* 2016) and Fréchet Inception Distance score (FID) (Heusel *et al* 2017) were adopted to evaluate whether the synthetic images and real images are in the same data distribution. The higher IS indicates better quality and diversity of the synthetic images. Moreover, the lower FID indicates a better image quality. The feature distribution similarity (FDS) was analyzed across of all real and synthetic data; if the synthetic images resemble the original images, these should be similar. We applied t-distributed stochastic neighbor embedding (T-SNE) (Van der Maaten and Hinton 2008) algorithm to embed each image into a two-point feature space to generated compressed features. Then we fit Gaussian models to the features from real and synthetic data, respectively. The FDS is measured by Kullback–Leibler divergence (KL) between the real and synthetic Gaussian distributions, where smaller KL indicates more similar distributions, therefore higher feature similarity and better quality.

Lastly, we calculated the diversity score (DS) to evaluate the diversity of the synthetic images. For each synthetic image, we calculated a structural similarity index measure (SSIM) between it and every other synthetic image to find the most similar pair. Then the SSIMs among all the synthetic images with their corresponding most similar pair are reported nearest SSIMs. We assume that if the real images have most of their nearest SSIMs in a specific range, the synthetic images should also have their nearest SSIMs concentrated in that same range. The new DS, or called nearest SSIM difference, is calculated using the KL divergence between the distribution of nearest SSIMs in real and synthetic images. This metric can be used to report the diversity of the synthetic images and indicate their similarity to the real images. Specifically, a low DS suggests that the synthetic images have a similar diversity to the real images.

Moreover, the proposed MT-DDPM is compared to widely-used image synthesis networks, including Wasserstein GAN with gradient penalty (WGAN) (Gulrajani *et al* 2017), Progressive-Growing GAN (PGGAN) (Segal *et al* 2021), Style GAN (SGAN) (Karras *et al* 2019), and Denoising diffusion probabilistic model (DDPM) (Ho *et al* 2020) by the quantitative evaluations in all datasets. The GAN-based model hyperparameters are carefully tuned for each evaluated image modality and their hyperparameters are shown in appendix A. The DDPM’s hyperparameters follows the proposed MT-DDPM.

### 4.4. Quantitative evaluation in a classification application

We validated the MT-DDPM framework’s effectiveness in medical-AI algorithm design by utilizing a COVID-19 classification network. He *et al* (2020) proposed a dense efficient network to diagnose COVID-19 based on lung CT images by utilizing a published dataset consisting of 349 positive and 397 negative cases. We aim to apply the MT-DDPM framework to generate synthetic data to evaluate whether the synthetic images can replace the real data for model training with a comparable performance or mix with real data to enhance the model classification performance. Their dataset was firstly resampled to a consistent resolution of $128\times 128$ for each image, then split into real-training (606 images) and real-testing data (146 images). The MT-DDPM framework will generate 1000 synthetic images with COVID-19 in the positive real-training cases and 1000 images without COVID-19 in the negative real-training cases. The classification network was trained, following the settings in He *et al*’s work (He *et al* 2020), individually on the real-training dataset (606 images), the synthesis dataset (2000), and a dataset mix of the real and synthetic images (2000 + 606 images). All the three models are tested on the real-testing dataset with accuracy, sensitivity, and specificity reported. Results of using only real training dataset are considered as baseline. We expect those quantitative results of only using synthesis dataset as training dataset to be comparable with baseline, and those of using mixed real and synthetic images as training dataset to be better than baseline. A McNemar’s test is performed to compare real versus synthetic dataset, and real versus mixed dataset, to evaluate the statistical significance between the network’s performance using different datasets.

### 4.5. Ablation study for hyper-settings

To thoroughly evaluate the performance of the MT-DDPM framework, we performed two ablation studies, one for diffusion settings and one for network settings using the abdomen CT dataset. We first evaluated the effect of different diffusion timestep $t$ on the quality of the synthetic images by comparing the FID, IS, intensity distribution, and diversity evaluation result when $T=4000,$ $T=2000,$ $T=1000,$ and $T=500.$ In addition, we performed the MT-DDPM with different number of filters in all the layers to validate the effect of the network’s total number of parameters on the synthetic images. Finally, the proposed MT-DDPM model were compared with $25 \% $ MT-DDPM (reducing the number of filters in each layer by 75%), $50 \% $ MT-DDPM (reducing $50 \% $ of number of filters in each layer), and $75 \% $ MT-DDPM (reducing $25 \% $ of number of filters in each layer). Furthermore, we report the computation efficiency (Training time of each epoch, inference time of each patient, and number of model parameter) in the different network settings.

## 5. Result

**Figure 2.**
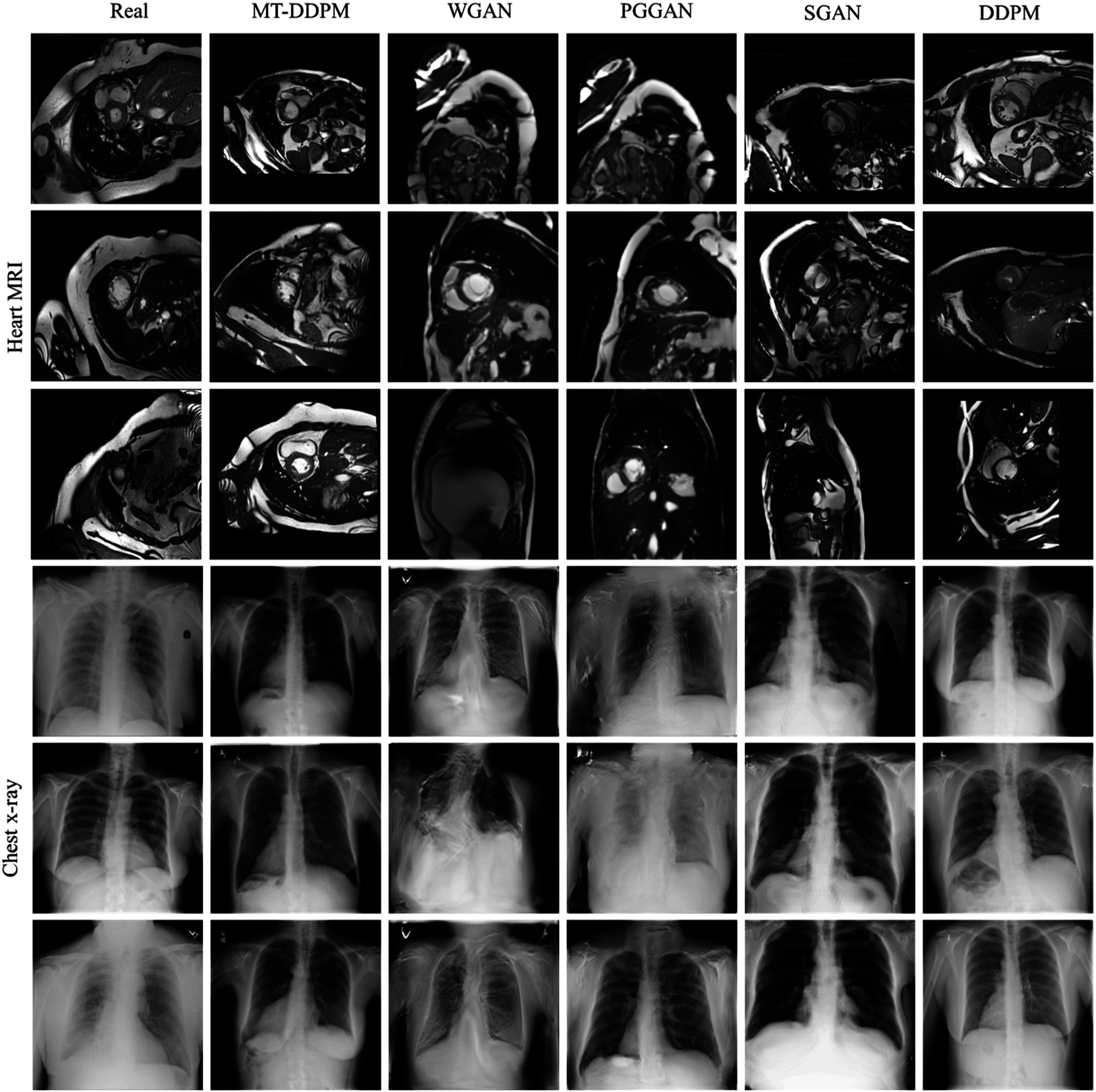
Synthetic and real images from chest x-rays, heart MRI. In each dataset, the real images, the synthetic images of the proposed MT-DDPM, WGAN, PGGAN, SGAN, and DDPM are presented in column-wise. Three slices of chest x-rays (row #1 to #3), heart MRIs (row #4 to #6) are displayed. Notice that for visualization purposes, the examples from different modalities were resized to the same resolution but could with different pixel spacings.

**Figure 3.**
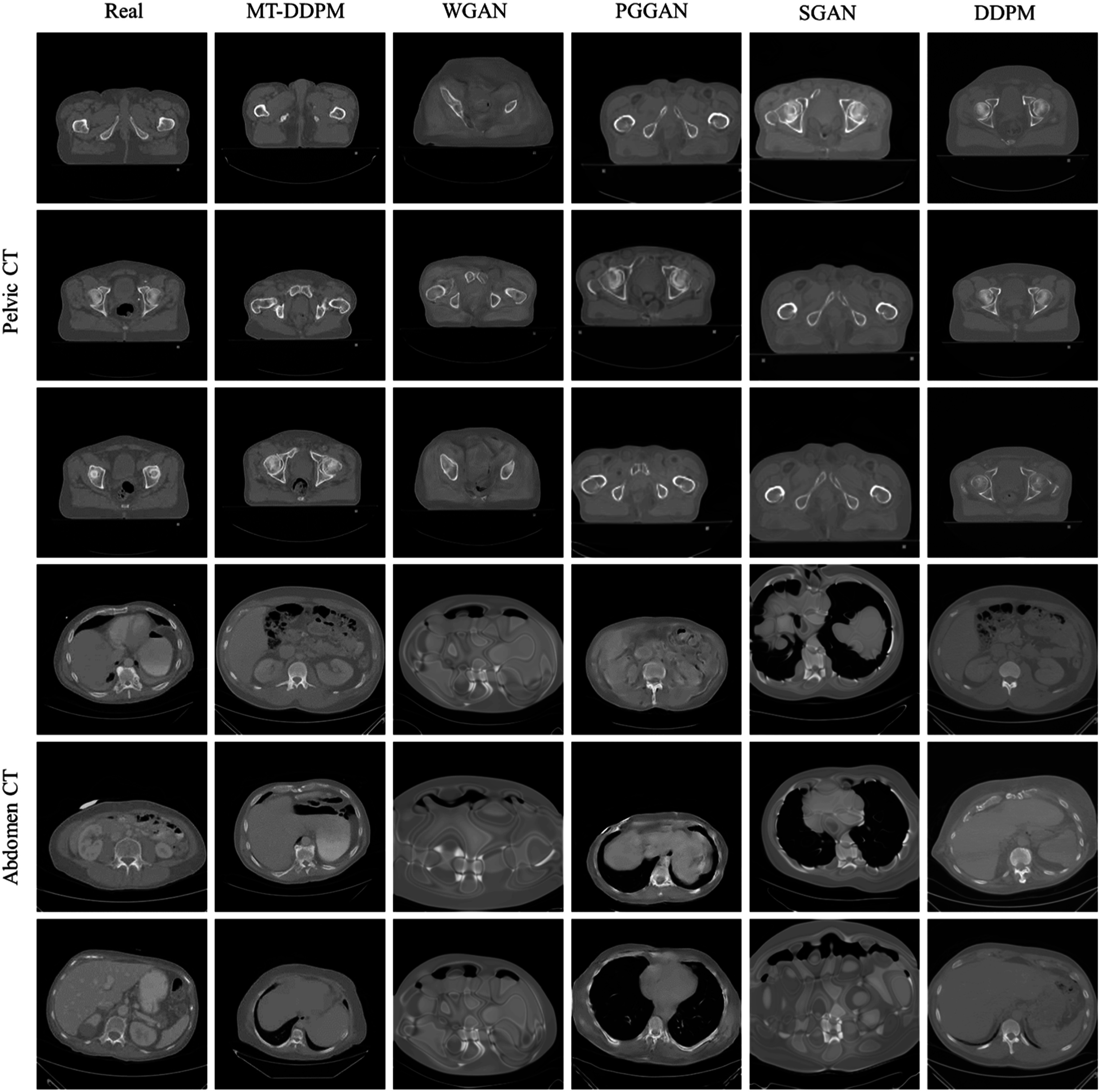
Synthetic and real images from pelvic and abdomen CT. In each dataset, the real images, the real images, the synthetic images of the proposed MT-DDPM, WGAN, PGGAN, SGAN, and DDPM are presented in column-wise. Three slices of pelvic CT images (row #1 to #3), and abdomen CT images (row #4 to #6) are displayed.

A visual comparison of the synthetic images from different imaging modalities is shown in figure 2. We present the visual Turing assessment in table 1. The results from the proposed MT-DDPM framework and the competing algorithms are summarized in table 2. In terms of the FDS evaluation, the corresponding T-SNE feature space visualization is shown in figure 3. The results of the application evaluation are presented in table 3. And the quantitative evaluation results for the ablation experiments in table 4.

**Table 1.** Statistic for the visual Turing assessment. The average accuracy, sensitivity, and specificity of three physicians are reported.

	Chest x-rays	Heart MRI	Pelvic CT	Abdomen CT
Accuracy	0.57	0.56	0.77	0.64
Sensitivity	0.70	0.64	0.97	0.83
specificity	0.48	0.45	0.59	0.46

**Table 2.** Quantitative analysis: table shows statistics for the FID, IS, IDS, and DS for the proposed MT-DDPM framework, WGAN, PGGAN, SGAN, and DDPM. (↑) means the higher value indicates better performance. (↓) means the lower value indicates better performance. The best results are bolded.

IS (↑)	Chest x-rays	Heart MRI	Pelvic CT	Abdomen CT
MT-DDPM	**2.07 ± 0.13**	**2.87 ± 0.16**	**2.07 ± 0.07**	**2.10 ± 0.12**
WGAN	2.05 **±** 0.09	2.31 **±** 0.11	1.77 **±** 0.08	1.91 **±** 0.06
PGGAN	2.06 **±** 0.05	2.32 **±** 0.10	1.97 **±** 0.06	1.99 **±** 0.06
SGAN	1.86 **±** 0.09	2.43 **±** 0.17	1.82 **±** 0.05	2.01 **±** 0.07
DDPM	2.04 **±** 0.10	2.64 **±** 0.14	2.00 **±** 0.10	2.02 **±** 0.14
FID (↓)	Chest x-rays	Heart MRI	Pelvic CT	Abdomen CT
MT-DDPM	**22.33**	**58.81**	**30.40**	**37.54**
WGAN	140.71	200.95	93.17	198.57
PGGAN	130.26	202.62	135.27	100.81
SGAN	182.80	240.86	100.62	170.05
DDPM	26.71	65.09	87.09	51.66
FDS (Feature KL) (↓)	Chest x-rays	Heart MRI	Pelvic CT	Abdomen CT
MT-DDPM	0.56	**0.15**	**0.03**	0.05
WGAN	2.82	2.96	0.77	1.50
PGGAN	**0.05**	3.06	2.29	**0.03**
SGAN	5.30	3.18	2.46	1.95
DDPM	0.73	0.31	2.77	0.10
DS (nearest SSIM diff) (↓)	Chest x-rays	Heart MRI	Pelvic CT	Abdomen CT
MT-DDPM	**0.29**	**0.94**	**1.84**	**0.36**
WGAN	0.95	6.32	2.44	2.94
PGGAN	1.26	1.19	17.26	1.19
SGAN	2.09	6.35	14.95	2.84
DDPM	1.11	2.07	5.24	0.70

**Table 3.** Quantitative evaluation in classification application. The performances of the baseline dense classification network trained on the real, MT-DDPM synthesis, and mixed datasets of real and MT-DDPM synthesis are presented.

	Real	MT-DDPM synthesis	Mix
Accuracy	0.88	0.89	0.93
Sensitivity	0.86	0.82	0.90
specificity	0.90	0.96	0.96
*P*-value	\	0.99	0.30

**Table 4.** Quantitative analysis for the ablation study with different model settings. The FID, IS, IDS and DS are presented for the proposed MT-DDPM framework with different diffusion steps in the first half of the table, including diffusion step $T=500,\,1000,\,2000,\,4000,$ where 4000 is the proposed setting. Then, the statistics of the evaluations results of the proposed MT-DDPM framework with different network parameters are shown, including $25 \% ,\,50 \% ,\,75 \% ,\,100 \% $ models, where the $100 \% $ model indicates the full network utilized in the MT-DDPM framework. (↑) means the higher value indicates better performance. (↓) means the lower value indicates better performance. The best results are bolded. The model efficiencies, training time (per epoch), inference time (per patient), and model parameters, are also presented.

	*T* = 500	*T* = 1000	*T* = 2000	*T* = 4000
IS (↑)	2.09 **±** 0.11	**2.28 ± 0.13**	2.25 **±** 0.08	2.10 **±** 0.12
FID (↓)	55.00	39.26	41.20	**37.54**
FDS (Feature KL) (↓)	0.19	0.19	0.10	**0.05**
DS (nearest SSIM diff) (↓)	0.48	0.38	0.37	**0.36**
Training time (in secs)	142.52			
Inference time (in secs)	13.07	26.13	52.26	104.53
Model parameter (in Million)	115.43			
	25%-model	50%-model	75%-model	100%-model
IS (↑)	1.32 **±** 0.17	**2.38 ± 0.09**	2.21 **±** 0.10	2.10 **±** 0.12
FID (↓)	390.94	202.10	76.76	**37.54**
FDS (Feature KL) (↓)	4.69	1.19	2.27	**0.05**
DS (nearest SSIM diff)) (↓)	0.62	0.42	**0.31**	0.36
Training time (in secs)	46.78	76.43	109.25	142.52
Inference time (in secs)	24.70	44.32	72.02	104.53
Model parameter (in Million)	7.24	28.32	65.11	115.43

### 5.1. Quantitative results for visual turing study

The average accuracy values for chest x-rays and heart MRIs are close to 0.5, the average sensitivity value is 0.65, and the average specificity value is 0.45. The result indicates that physicists had difficulty identifying real or synthetic images. Accordingly, the synthetic images contain indistinguishable visual appearance to the real images.

The average accuracies for pelvic and abdomen CT images are around 0.75 and 0.65. With relatively high sensitivities, the specificities are still close to 0.5. Physicists can confidently recognize real images but still have great difficulties recognizing synthetic ones.

### 5.2. Quantitative results for the MT-DDPM and competing networks

Regarding two comprehensive evaluations, the MT-DDPM obtained the highest IS among all competing algorithms from all datasets. On one hand, the highest IS indicates that the MT-DDPM’s images have the best quality and diversity among the competing networks. It also obtains the lowest FID between the real and synthetic images and the from all evaluated datasets. The lowest FID demonstrates that the synthetic images from the MT-DDPM framework have the most similar InceptionV3 features’ distribution when compared to the real images, therefore, maintain more realistic visual characteristics than the WGAN and PGGAN.

**Figure 4.**
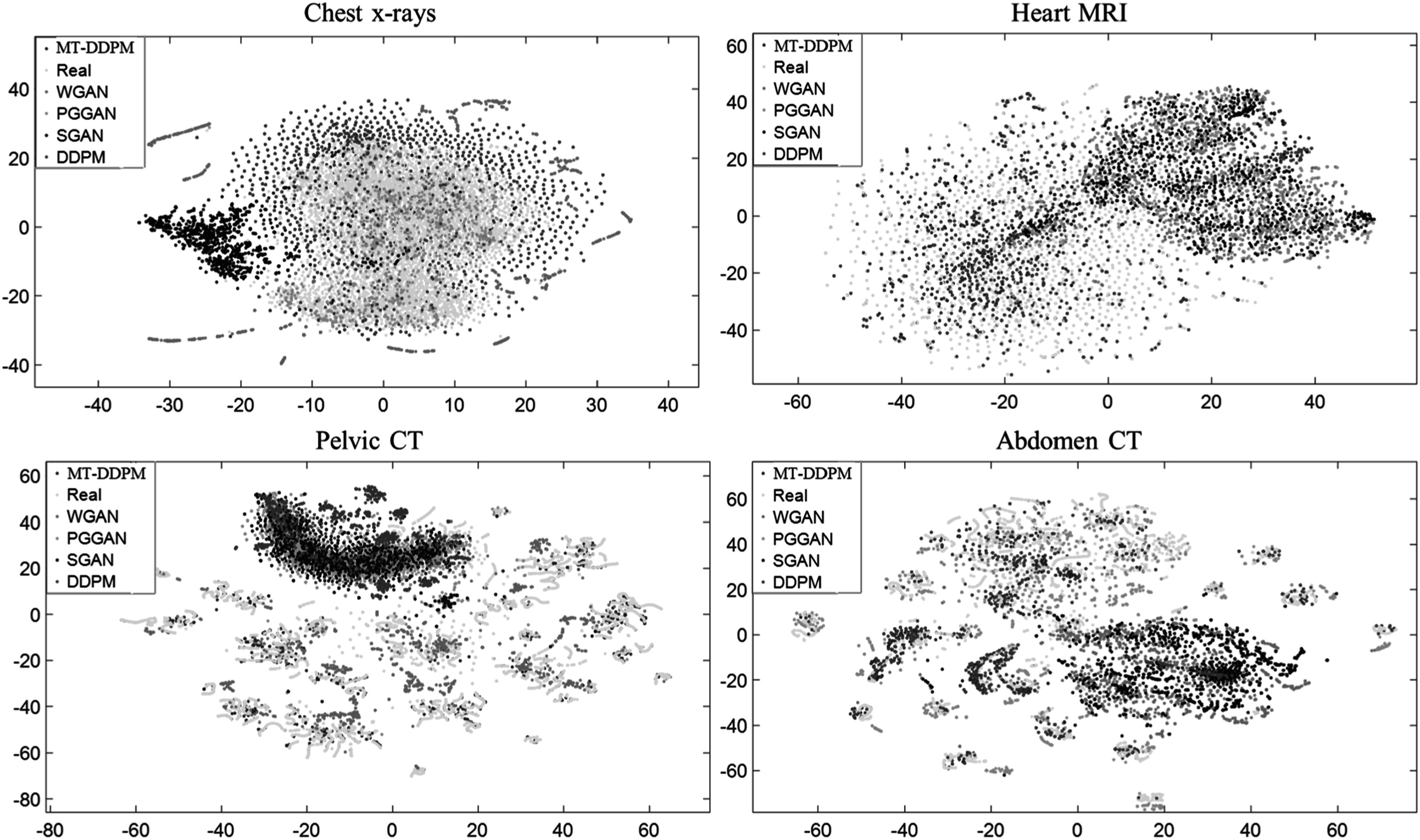
T-SNE feature space visualization for the MT-DDPM ’s synthetic image. The real features (yellow), MT-DDPM features (red), WGAN features (green), PGGAN features (blue), SGAN features (deep blue), and DDPM features (cyan purple) are shown for the chest x-rays (top left), heart MRI (top right), pelvic CT image (bottom left), and abdomen CT image (bottom right). We fit Gaussian model to each distribution and measure their distance by KL divergence as our FDS.

**Figure 5.**
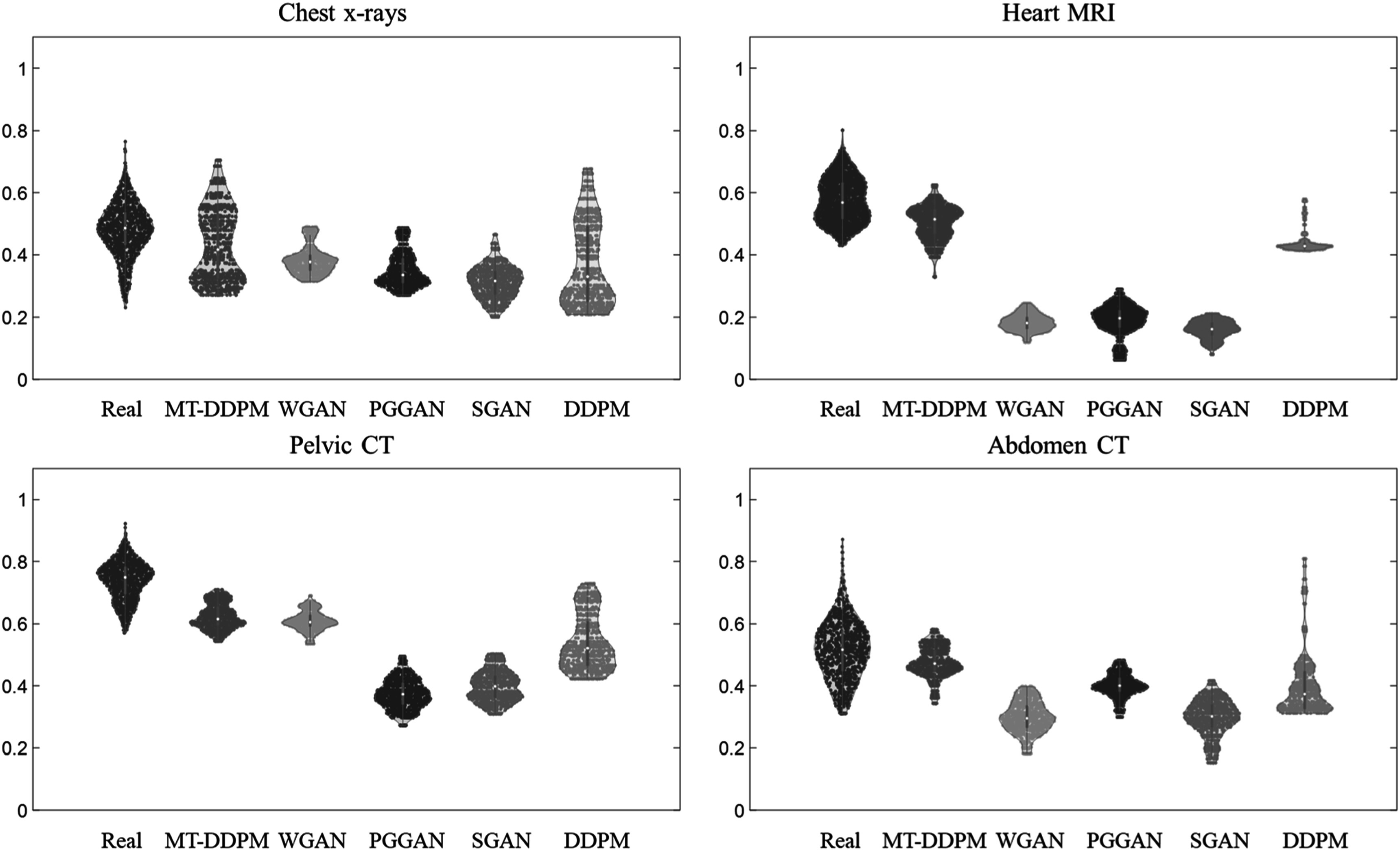
Violin plot of the SSIM between each synthetic image with its corresponding most similar pair. The DS (nearest SSIM) of the real image, synthetic images of the MT-DDPM, WGAN, PGGAN, SGAN, DDPM are shown for the chest x-rays (top left), heart MRI (top right), pelvic CT image (bottom left), and abdomen CT image (bottom right). Within each violin, the colored dots represent the SSIM for all sample pairs. The wings display the distribution of the SSIM; the thick gray bar displays the interquartile range; and the white dot inside the gray bar is the median of the SSIMs.

Regarding the feature comparison (visualization are shown in figure 4 and 5), the feature distribution of the MT-DDPM’s synthetic images has the lowest KL divergence to the features of real images in the heart MRI, pelvic, and abdomen CT datasets. In addition, the MT-DDPM’s synthetic images have the lowest DS in all modalities, which indicates that the MT-DDPM obtains the most similar nearest SSIMs (diversity) to the real images than the competing networks.

### 5.3. Quantitative evaluation in a classification application

The baseline dense network for COVID-19 achieves quantitively higher accuracy and specificity when using the pure synthetic dataset, compared to the network trained by the real-training dataset. The McNemar’s Test’s significance *p*-value was set at 0.05. Considering the MT-DDPM framework can generate medical data with greatly higher efficiency and less cost, the proposed MT-DDPM can still achieve statistically comparable classification performance to the real-world data in this task.

The network trained on the mixed dataset achieves the highest accuracy, sensitivity, and specificity compared to those trained on only the real or synthetic datasets. Although the *p*-value (>0.05) did not show statistical significance on the mixed dataset compared to the real dataset, the synthetic images can be considered reliable data augmentation to the real dataset, improving the network’s learning ability quantitatively.

### 5.4. Quantitative analysis in the ablation study

In terms of the diffusion step $T,$ the network demonstrated the best IS when $T=1000,$ FID, FDS, and DS when $T=4000.$ However, there are no obvious differences among metrics when $T=1000,\,2000,\,4000.$ Notice that the training time and number of model parameters are the same to the different $T.$ As a result, raising the number of training steps $T$ can enhance image synthesis performance. However, this also leads to increased computation time for inference.

In terms of model parameters, the proposed $100 \% $ model gained the best FID and FDS. Especially, for FID, we can observe a consistent improving trend with more network parameters. The $50 \% $ model obtains the best IS, and the $75 \% $ model gains the best DS. Except for the $25 \% $ model, there are no obvious differences among the other networks for IS, FID, and FDS. In summary, increasing MT-DDPM network’s parameter could be a reliable way to improve FID. And designing a MT-DDPM network with sufficient parameters could be necessary for maintaining high IS and FDS. Nonetheless, having a greater number of model parameters will also lead to longer training and inference times, and necessitates more memory resources for computation.

## 6. Discussion

In this work, we propose a Transformer-based Diffusion Model for 2D image synthesis. The proposed MT-DDPM framework has demonstrated the promising quality of its synthetic images, which can potentially be used as training dataset in DL model for specific tasks. Using synthetic images can reduce the effort required to collect, pre-process, store, and transfer large patient datasets, and is beneficial to small institutions, clinics, and research groups looking to develop and implement DL models with less data requirements.

The framework consists of two main components: a diffusion process and an MT-DDPM network. The diffusion process is composed of a forward process and a reverse process. The forward diffusion process adds Gaussian noise in multiple timesteps to the images from existing datasets in order to generate noisy images. The reverse process then uses a neural network (in this case, the MT-DDPM network) to learn to iteratively denoise the noisy images back to the noise-free images. Specifically, the diffusion reverse process defines denoising as a Gaussian Markov process with two parameters: mean and variance. The MT-DDPM network is first trained to estimate the accumulated noise added at timestep t, which allows it to derive the mean of the denoising process. The MT-DDPM network is also required to predict a variance interpolation coefficient, which is used to derive the variance. This allows the denoising process to iteratively transform a new pure Gaussian noise image into a new synthetic image with a realistic visual appearance. In terms of network design, the MT-DDPM network follows a U-shape encoder–decoder architecture. The encoder down-samples the input images to learn features in different resolution levels, while the decoder up-samples the features to the original input size to estimate the noise and the variance interpolation coefficient. Convolutional layers are deployed in the high-resolution levels to efficiently capture local information, while Swin-transformer layers, which take advantage of self-attention mechanisms, are set to low-resolution levels to model global-level characteristics and refine the network’s performance. To the best of our knowledge, this is the first diffusion model for medical image synthesis that can be applied to multiple imaging modalities. It is also the first attempt to utilize Swin-transformer in the design of a diffusion model to enhance the image synthesis quality.

The MT-DDPM framework was evaluated by a visual Turing assessment (table 1), quantitative evaluations (table 2), and an application evaluation (table 3). The MT-DDPM framework was able to generate synthetic images that were highly indistinguishable from real chest x-rays and heart MRI slice. Moreover, it can generate realistic pelvic and abdomen CT 2D slices. MT-DDPM outperformed other competing networks regarding image quality as measured by IS, FID, and FDS across most datasets. Notice that the IS and FID are evaluated by using an InceptionV3-Net was pre-trained on ImageNet dataset, which is a dataset that is not specific to medical imaging. Therefore, the InceptionV3-Net could potentially extract misleading information and, accordingly, is detrimental to the measurement ability of the IS and FID. However, many works [48, 49, 51, 52] have demonstrated that the FID is among the best quantitative evaluation correlated with human visual Turing tests for Abdomen CT images, Chest x-rays, and heart MRI synthesis. Therefore, the FID could be a strong indicator of the visual quality of our synthetic images. Moreover, while the FID cannot evaluate the diversity of the synthetic image, the IS can overcome this issue. In summary, the best IS and FID from the proposed MT-DDPM indicate that the network can achieve the best quality considering the image’s visual quality and diversity comprehensively. Furthermore, it achieved the highest FDS in the heart MRI and pelvic CT dataset, and DS in all the evaluated datasets, which indicates that the MT-DDPM obtains the most similar diversity distribution with the real images in all modalities. Although the MT-DDPM has slightly less diversity than some other image generating models that use GAN-based models, we can still conclude that the synthetic images generated by the MT-DDPM have an acceptable level of diversity. This is because the real images used as a basis for comparison should have a sufficient level of diversity, and the MT-DDPM’s synthetic images are more similar to the real images than the images generated by other GAN-based methods.

In the application evaluation, the MT-DDPM synthetic data achieved lower but statistically comparable quantitative performance compared to the real images. It was also able to be used as new way of data augmentation to enrich the existing real dataset and improve the training of the application network quantitatively. To summarize, the MT-DDPM framework produces visually appealing synthetic images with decent diversity. It outperforms commonly used existing generative models such as WGAN, PGGAN, SGAN, and DDPM, achieving state-of-the-art image quality and diversity.

In addition, ablation studies in section 4.5 shows that: (1) increasing training diffusion steps could slightly improve the abdomen image synthesis; (2) increasing network parameters could improve the abdomen image synthesis. Moreover, the GAN-based methods are very sensitive to their hyper-parameters: the hyper-parameters (shown in appendix A) must be carefully fine-tuned using excessive time and computing resources for each imaging modality to generate acceptable synthetic images. On the contrary, the proposed MT-DDPM and DDPM are less sensitive to their hyper-parameters and much more stable. Furthermore, the MT-DDPM can be easily implemented in x-rays, MRI, and CT to generate high-quality images, given by sufficient large network capacity and diffusion steps (shown in the ablation study).

Despite these gains, the MT-DDPM framework’s relatively low efficiency in generating synthetic images is worth noting. With the same hardware configuration shown in section 4.1, generating a resolution image took approximately 220 s for the MT-DDPM framework (maximum timestep is 4000). In contrast, it takes only 0.18 s for the WGAN and PGGAN to generate the image with the same resolution. In addition, the MT-DDPM network has much higher complexity than the GAN-based algorithms. The proposed network contains $1.6\times {10}^{9}$ parameters, while the WGAN and PGGAN contain a total of parameters $8.6\times {10}^{7}$ for both the discriminator and generator. These limitations prevented us from extending the existing idea into 3D volume synthesis, as the time and complexity required for a 3D diffusion framework will be prohibitively expensive. However, these limitations do not diminish the value of the MT-DDPM framework but rather highlight the need for further improvement in its efficiency. Zhang and Chen (2022) proposed a diffusion model for image synthesis with as few as ten timesteps by designing an exponential forward process to generate noisy images. Kong and Ping (2021) deployed a pre-trained network to accelerate the image synthesis process with much fewer diffusion timesteps. These types of algorithms could be a future direction to improve the MT-DDPM framework’s efficiency and enable 2D image synthesis to be extended to 3D medical volume synthesis.

In the future, we plan to incorporate advanced techniques to support 3D medical volume synthesis, which are more commonly-used image domain for MRI and CT images. We will work on improving the efficiency of the diffusion framework for the 3D synthesis. In addition, we will investigate and validate the ability of the diffusion framework to generate 3D volumes. Furthermore, we will explore more sophisticated network architectures to improve image synthesis quality. We will also develop a deterministic MT-DDPM framework for medical volume-to-volume translation (e.g., MRI to CT translation). We intend to conduct a broader study of the proposed MT-DDPM in various applications beyond classification, including organ segmentation and tumor detection. Human experts will participate by providing ground truth organ contours or tumor location on synthetic images to evaluate the effectiveness of MT-DDPM in these applications.

## 7. Conclusion

This work presents a MT-DDPM-based medical image synthesis framework that utilizes a Swin-transformer-based network to learn a diffusion model to generate synthetic images. The proposed MT-DDPM is able to generate medical images with a realistic visual appearance similar to real medical images. It is also able to capture similar characteristics to the real images with high diversity. The MT-DDPM can be a reliable tool to generate high-quality synthetic data to support data-driven medical applications.

## Data Availability

The data cannot be made publicly available upon publication because they contain sensitive personal information. The data that support the findings of this study are available upon reasonable request from the authors.

## Conflict of interest

The authors have no conflict of interests to disclose.

## Ethical statement

Emory IRB review board approval was obtained (IRB #114349), and informed consent was not required for this Health Insurance Portability and Accountability Act (HIPAA) compliant retrospective analysis.
